# Evaluation of the performance of the IFN-γ release assay in bovine tuberculosis free herds from five European countries

**DOI:** 10.1186/s13567-023-01187-5

**Published:** 2023-07-04

**Authors:** Alberto Gomez-Buendia, Beatriz Romero, Javier Bezos, José Luis Saez, Ivonne Archetti, Maria Lodovica Pacciarini, Maria Laura Boschiroli, Sébastien Girard, Emanuela Gutu, Florica Barbuceanu, Ourania Karaoulani, Athanasia Stournara, Lucia de Juan, Julio Alvarez

**Affiliations:** 1 VISAVET Health Surveillance Centre, Universidad Complutense de Madrid, Madrid, Spain; 2 Departamento de Sanidad Animal, Facultad de Veterinaria, Universidad Complutense de Madrid, Madrid, Spain; 3 Subdirección General de Sanidad e Higiene Animal y Trazabilidad, Dirección General de la Producción Agraria, Ministerio de Agricultura, Pesca y Alimentación, Madrid, Spain; 4 National Reference Centre for Bovine Tuberculosis, Istituto Zooprofilattico Sperimentale della Lombardia e dell’Emilia Romagna, Brescia, Italy; 5 University Paris-Est, Laboratory for Animal Health, Tuberculosis National Reference Laboratory, ANSES, Maisons-Alfort, France; 6 Regional Directorate for Food, Agriculture and Forest of Bourgogne-Franche-Comte, Dijon, France; 7 Institute for Diagnosis and Animal Health, Bucharest, Romania; 8 National Reference Laboratory for Bovine Tuberculosis, Directorate of Veterinary Centre of Athens, Ministry of Rural Development and Food, Athens, Greece; 9 Department of Serology, Veterinary Laboratory of Larissa, Ministry of Rural Development and Food, Larissa, Greece

**Keywords:** Bovine tuberculosis, interferon-gamma release assay, specificity, Bayesian statistics, cut-off, *Mycobacterium tuberculosis* complex

## Abstract

The diagnostic methods for granting and maintenance of the official tuberculosis-free (OTF) status and for intra-Community movement of cattle are the tuberculin skin tests (single or comparative) and the interferon-γ (IFN-γ) release assay (IGRA). However, until now, IGRAs have been primarily applied in infected farms in parallel to the skin test to maximize the number of infected animals detected. Therefore, an evaluation of the performance of IGRAs in OTF herds to assess whether if their specificity is equal to or higher than that of the skin tests is needed. For this, a panel of 4365 plasma samples coming from 84 OTF herds in six European regions (five countries) was assembled and analysed using two IGRA kits, the ID Screen^®^ Ruminant IFN-g (IDvet) and the Bovigam™ TB Kit (Bovigam). Results were evaluated using different cut-offs, and the impact of herd and animal-level factors on the probability of positivity was assessed using hierarchical Bayesian multivariable logistic regression models. The percentage of reactors ranged from 1.7 to 21.0% (IDvet: S/P ≥ 35%), and 2.1–26.3% (Bovigam: OD_bovis_–OD_PBS_ ≥ 0.1 and OD_bovis_–OD_avium_ ≥ 0.1) depending on the region, with Bovigam disclosing more reactors in all regions. The results suggest that specificity of IGRAs can be influenced by the production type, age and region of origin of the animals. Changes in the cut-offs could lead to specificity values above 98–99% in certain OTF populations, but no single cut-off yielding a sufficiently high specificity (equal or higher than that of skin tests) in all populations was identified. Therefore, an exploratory analysis of the baseline IFN-γ reactivity in OTF populations could help to assess the usefulness of this technique when applied for the purpose of maintaining OTF status.

**Supplementary Information:**

The online version contains supplementary material available at 10.1186/s13567-023-01187-5.

## Introduction

Animal tuberculosis is a worldwide zoonotic disease included in the WOAH list of notifiable diseases caused by *Mycobacterium tuberculosis* complex (MTBC) members [1]. From 2019 to 2022, around 158 countries took measures to prevent animal TB, and 62 of them applied a “test and cull” strategy on their cattle population [2]. This disease affects not only cattle, its main host in most countries, causing bovine tuberculosis (bTB), but also a wide variety of species, both domestic and wild [3, 4]. In the European Union (EU), based on Annex III to Delegated Regulation (EU) 2020/689 and Annex I to Delegated Regulation (EU) 2020/688 of the Regulation (UE) 2016/429, the intradermal tuberculin tests and interferon (IFN)-γ release assay (IGRA) are the official tests for granting and maintenance of the official TB-free (OTF) herd status and to obtain the certification for intra-Community trade of animals.

The IGRA was first introduced in the EU legislation in 2002 for the purpose of maximizing the number of infected animals detected when used in parallel with the skin test [Commission Regulation (EC) No. 1226/2002 of 8 July 2002 amending Annex B to Council Directive 64/432/EEC, both derogated to date]. Since then, several studies have assessed its performance in infected herds, with specificity values (based on Bayesian latent class models) ranging between 62 and 98% (depending on the kit and cut-off applied) [5–12]. In contrast, fewer studies in OTF herds have been performed, suggesting a specificity from 83 to 99% based on the assumption that all reactors were false-positive animals [13–17], and indicating that the IGRA might be a good candidate to be applied under OTF conditions at least in certain cases. However, because different cut-off points, interpretation criteria, kits, and protocols (e.g., time between sample collection and stimulation) were used, comparisons between study results should be interpreted with care.

Given the potential of local factors to influence the specificity of bTB diagnostic tests [15, 18], additional information on the performance of the IGRA test in OTF populations is needed to optimize its use and assess the impact of different cut-off values in the context of maintaining OTF status. For this purpose, an IGRA should ideally offer a specificity not lower than that of the standard test (single or comparative skin tests) while maintaining an adequate (i.e., not lower than that of the standard) sensitivity as specified by the EFSA [19]. However, current estimates of the sensitivity and specificity of IGRAs have been obtained through a range of protocols based on different antigens, subjected to the possible booster effect of a previous skin test, variable times between collection and stimulation of blood samples, different cut-offs, commercial kits, and tests were assayed in different animal populations (in terms of e.g., age, breed, production type, herd size, presence of non-tuberculous mycobacteria (NTM), region, etc.). All these factors may influence the performance of IGRAs [20–25]. Therefore, the assessment of the performance of IGRA tests following harmonised protocols and taking into consideration the potential effect of individual and herd level factors is still needed to assess its suitability for the purpose of granting and maintenance of the OTF status and movement of cattle within the EU [19].

Here, a large panel of samples from five EU countries was assembled and tested using two different IGRA kits, the ID Screen^®^ Ruminant IFN-g (IDvet) and the Bovigam™ TB Kit (Bovigam), in order to i) evaluate the performance of IGRAs under different epidemiological conditions in bovine tuberculosis-free herds with a view to assess its usefulness for granting and maintenance of the OTF status of herds and the intra-Community trade of animals, and ii) assess the impact of different cut-off values in both kits.

## Materials and methods

### IFN-γ release assay

A panel of 4365 plasma samples coming from six regions (A-F) located in five EU countries (France, Greece, Italy, Romania and Spain) was collected by local authorities and analysed at the European Union Reference Laboratory (EU-RL) for Bovine Tuberculosis located in the Veterinary Health Surveillance Centre (VISAVET) of the Complutense University of Madrid.

Blood samples were collected from OTF herds of animals at least 6 months old. In two regions (B and F) certain OTF herds in which non-specific reactions to the skin test (attributed to the presence of NTM) had previously been described were intentionally included in the study. Samples were collected in heparinized tubes at least 4 months after the previous skin test and transported and stimulated to a laboratory in each of the regions within eight hours post collection. Also, a single skin test was performed the same day the blood sample was collected. Blood from each animal was distributed in four wells of a 24-well plate and stimulated with PBS, avian purified protein derivative (PPDa) (CZ Veterinaria, Porriño, Spain) (20 µg/mL), bovine PPD (PPDb) (CZ Veterinaria, Porriño, Spain) (20 µg/mL) and pokeweed mitogen (Lectin from *Phytolacca americana*, Sigma, Merck KGaA, Darmstadt, Germany) (2 µg/mL), included as a measure of lymphocyte viability [26, 27]. All antigens and PBS belonging to the same batch were provided by the EU-RL. Plates were then incubated for 18–24 h at 37 °C in a humid atmosphere and then centrifuged at 500–770 *g* for 10–15 min. Around 400–500 µL of plasma was collected from each well, frozen and shipped to the EU-RL for further analysis.

Plasma-stimulated samples were then analysed for the presence of IFN-γ using the IDvet (ID Screen^®^ Ruminant IFN-γ, IDvet, Innovative Diagnostics, Gravels, France) and Bovigam (Bovigam™ TB Kit, Thermo Fisher Scientific, Waltham, MA, USA) kits in the same day according to the manufacturer instructions (using 25 µL of each sample + 25 µL of dilution buffer 1 for IDvet, and 50 µL of each sample + 50 µL of Green Diluent for Bovigam). Results were expressed as optical densities (OD) by reading the absorbance of each well at 450 nm for IDvet, and at 450 nm with a reference of 620 nm for Bovigam.

For the qualitative interpretation of the Bovigam test two values were considered, the OD of the bovine-stimulated sample (OD_bovis_) minus the OD of the PBS-stimulated sample (OD_PBS_), and the OD_bovis_ value minus the OD of the avian-stimulated sample (OD_avium_).

In the case of the IDvet test, results were transformed to a sample-to-positive (S/P) ratio considering the values of the positive and negative controls included in each plate as follows:

$$S/P = \left( { \frac{OD\,bovis - OD\,avium}{{OD\,mean\,positive\,control - OD\,mean\,negative\,control}} } \right) \times 100$$

Cut-offs recommended by the manufactures (Table 1) were initially used for interpretation of the quantitative outcomes of the assays.

**Table 1:** **Cut-off points used for the IFN-γ ELISA assays**

Test	Cut-off	Result	Sample
IDvet	S/P ≥ 35%	Negative	S/P < 35%
		Positive	S/P ≥ 35%
		Non-valid	S/P_mitogen_ < 35% or OD_PBS_ > 2.5 or
			OD_bovis_ and OD_avium_ > 2.5
Bovigam	OD_bovis_–OD_PBS_ ≥ 0.1 and OD_bovis_–OD_avium_ ≥ 0.1	Negative	OD_bovis_–OD_PBS_ < 0.1 or
			OD_bovis_–OD_avium_ < 0.1 or
			OD_bovis_–OD_PBS_ ≥ 0.1 and OD_bovis_–OD_avium_ < 0.1 or
			OD_bovis_–OD_PBS_ < 0.1 and OD_bovis_–OD_avium_ ≥ 0.1
		Positive	OD_bovis_–OD_PBS_ ≥ 0.1 and OD_bovis_–OD_avium_ ≥ 0.1
		Non-valid	OD_mitogen_–OD_PBS_ < 0.1 or OD_PBS_ > 3.5 or
			OD_bovis_ and OD_avium_ > 3.5

In addition, each of the plates were validated considering the following criteria: for IDvet, the mean OD value of the positive controls had to be greater than 0.5 and higher than three times the mean OD value of the negative controls; for Bovigam, the mean OD value of the negative controls had to be below 0.130 with a maximum difference of 0.040 between them, and the mean OD value of the positive controls greater than 0.7 with a maximum difference between them of 30% of their mean value.

### Statistical analysis

From each sample, information on the age, region of origin (A–F), production type (beef or dairy), result of the previous cervical skin tests (single or comparative) and the one performed the sampling day (in millimeters), and herd size was available. Previous skin test results could be negative, single-inconclusive [PPDb skin fold increase of ≥ 3 mm but lower than the PPDa skin fold increase and without clinical signs in the inoculation site and therefore negative in the comparative skin test depending on whether herds were subjected to single or comparative skin testing (EU-RL Standard Operating Procedure SOP/001/EURL)]. In addition, for animals coming from regions B and F data on the history of presence of NTM or *M. avium* subsp. *paratuberculosis* (MAP) was collected. Also, 510/512 animals in region C were tested using a paratuberculosis (PTB) serology test (ID Screen^®^ Paratuberculosis Indirect ELISA, IDvet, Innovative Diagnostics, Gravels, France).

All statistical analyses were performed in R [28] except where indicated. The proportion of reactors using the default cut-offs was calculated for each test using the default cut-off points (Table 1). The agreement between tests was assessed using the Kappa statistic, the proportion of reactors in each test was compared using the McNemar test and the differences of age between production type was assessed using a Student's *t*-test. In addition, the quantitative results obtained in the IDvet (S/P ratio) and Bovigam (OD_bovis_–OD_avium_) were compared using Pearson’s correlation coefficient.

Then, receiver operating characteristic (ROC) curves were used to evaluate the performance of the IDvet kit at different cut-offs in relation to the qualitative results of the Bovigam with the default cut-off and vice versa. The first analysis (quantitative IDvet results in relation to qualitative Bovigam results) was performed using the R package “pROC” [29]. Confidence intervals (CI) and the optimal cut-off point for the ROC curve was estimated through 1000 bootstrap replicates using the package “cutpointr” [30].

The second analysis (quantitative Bovigam results in relation to qualitative IDvet results) was performed using the package “Epi” [31] to allow the use of two predictors (OD_bovis_–OD_PBS_ and OD_bovis_–OD_avium_) when estimating the ROC curve. Optimal cut-off points were calculated based on the formula:

$$outcome = \frac{1}{{1 + e^{ - \left( {\beta_0 + \beta_1 X_1 + \beta_2 X_2 } \right)} }}$$

where “outcome” is the best logistic regression estimate for the optimal cut-off points, *β*_0_ is the intercept of the model, *β*_1_ and *β*_2_ are the coefficients of the predictors, and *X*_1_ and *X*_2_ the values of the predictors itself.

Finally, the probability of yielding a positive result in the IGRA depending on the effect of the available covariables was evaluated for each kit separately through a Bayesian multivariable logistic regression model of the form:

$$\begin{aligned} Z_{ij} \sim Bernouilli\left( {p_{ij} } \right) \\ logit\left( {p_{ij} } \right) = \alpha_j + \beta_1 X_{ij1} + \beta_2 X_{ij2} + \cdots + \beta_k X_{ijk} \\ \end{aligned}$$

where Z_i,j_ is the test result (negative/positive) of animal *i* from herd *j*, *p*_*ij*_ is the probability that this animal tests positive, *α*_*j*_ is the herd-level effect for herd *j*, *β*_1_, …, *β*_*k*_ are the coefficients of the covariables at the animal level, and *X*_1_, …, *X*_*k*_ the values of those covariables.

The herd-level effect was then assumed to follow a normal distribution as follows:

$$\begin{aligned} \alpha_j \sim N\left( {\mu_j ,\sigma_{herd} } \right) \\ \mu_j = \delta_0 + \delta_1 Y_{j1} + \delta_2 Y_{j2} + \cdots + \delta_l Y_{jl} \\ \end{aligned}$$

where *δ*_1_, …, * δ*_*l*_ are the coefficients of the covariables assessed at herd level and *Y*_1_, …, *Y*_*l*_ the values of those covariables.

The covariables used at the animal level included the age (available for all animals) and the result of the animal at previous skin tests. The region of origin of the herd, the production type, the herd size and the information on presence of PTB and/or NTM in the herd (yes/no, assuming that animals from herds in which no information on the presence of PTB/NTM was available were not exposed to these bacteria) were included at herd level.

Age and herd size were evaluated alternatively as continuous and categorical variables. For age, four categories were considered: < 1 year, 1–4 years, 4–7 years, and more than 7 years. Herds were categorized based on their size on herds with < 30 animals, 30–59, 60–100, and more than 100 animals.

Samples from region C were subjected to a separate analysis in which the individual result obtained in the PTB serological test was also added as a covariate at the animal level following the same model.

Weakly informative Normal (0, 1) priors were used for the $\beta$ and $\delta$ coefficients. Herd-level random effects (α) were assumed to follow a Normal (μ, σ^2^) distribution, with σ ~Uniform (0, 1). The best model was selected based on the lowest DIC (Deviance Information Criteria) [32].

Models were fitted in WinBUGS [33] through the R package “R2WinBUGS” [34]. Three Markov Chain Monte Carlo chains were run for 10 000 iterations, with a “burn-in” of 1000 iterations, and posterior distributions were obtained after thinning every 10 iterations. Convergence was assessed visually and more formally using the Gelman-Rubin statistic [35].

Finally, the percentage of reactors at alternative cut-off points within justifiable ranges (S/P ≥ 15–120% range for IDvet, and OD_bovis_–OD_PBS_ ≥ 0.01–1.0 and OD_bovis_–OD_avium_ ≥ 0.01–1.0 range for Bovigam) based on the observed quantitative results was assessed to evaluate such thresholds on different populations.

## Results

### Population of study

All plates were validated according to the manufacturers’ instructions. Out of the 4365 samples received, nine were discarded because there was insufficient volume, and 54 and 49 (~1.5%) yielded non-valid results in the sample stimulated with mitogen when analysed with the IDvet and Bovigam assays, respectively (46 were non-valid in both tests).

Therefore, a total of 4299 samples with results for both tests were included in the study. Animals originated from 84 herds (mean = 51.2 animals per herd, median = 31, range = 5–248), with regions contributing with between 376 and 1225 samples from between 3 and 45 herds (Table 2). All regions included samples from dairy cattle, while beef cattle was not available in regions D and E (Table 2).

**Table 2:** **Distribution of the population under study**

Region	Total animals	Total herds	Dairy		Beef	
			Animals	Herds	Animals	Herds
A	1225	11	649	5	576	6
B	1202	45	607	9	595	36
C	512	8	112	2	400	6
D	495	3	498	3	–	–
E	376	5	378	5	–	–
F	489	12	380	10	109	2
Total	4299	84	2619	34	1680	50

**Figure 1:**
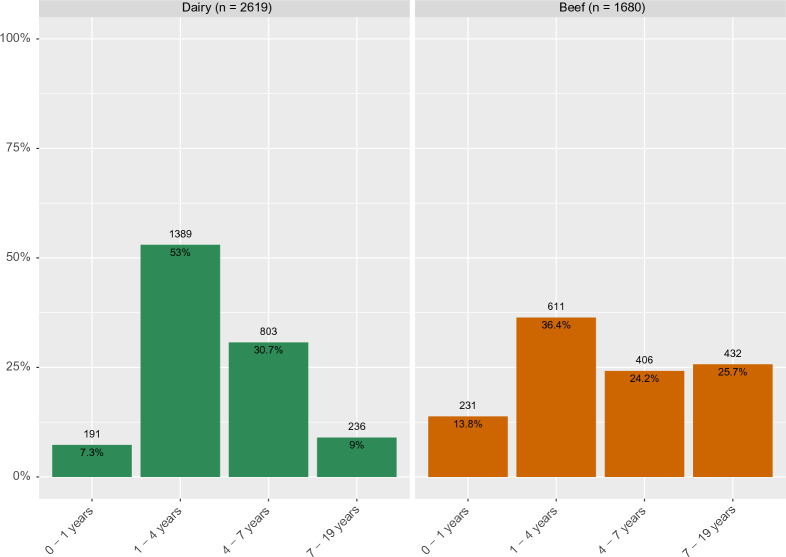
**Age of the sampled animals (*n*** **= 4299) by production type**

Mean age of sampled animals was 4.2 years (median = 3.6, range = 0.5–18.9), with beef cattle being significantly (Student's *t*-test, *p* < 0.001) older (mean = 5.0 years, median = 4.0, range = 0.5–18.9) compared to dairy cattle (mean = 3.8, median = 3.5, range = 0.5–15.9 years) (Figure 1).

Regarding the exposure to other mycobacteria, MAP had been isolated in three and one herds located in regions B (out of 45 herds) and F (out of 12 herds), respectively, and other NTM had been also recovered from cattle located in five herds from region B (in one both MAP and NTM were recovered).

Furthermore, 10 animals from three herds from region C tested positive to the PTB ELISA. Finally, although no reactors were found in the skin test performed when the blood samples were collected, 44/1202 animals from 16/45 herds in region B were comparative-inconclusive, and two out of 489 animals from one herd in region F were single-inconclusive on a previous testing.

### Qualitative results using reference cut-off points

A larger proportion of reactors was observed when the Bovigam kit was used compared with the IDvet regardless of the region, production type or age category (overall proportion of reactors in Bovigam 9.8% vs. 7.3% in IDvet, Table 3). Also, there were more herds with at least one positive to Bovigam (60/84 herds; 71.4%) than to IDvet (49/84; 58.3%).

**Table 3:** **Number of reactors in both kits divided by region, production type and age interval**

Variable	Population		Positive Bovigam^a^		Positive IDvet^b^	
	Animals	Herds	Animals	Herds	Animals	Herds
Region						
A	1225	11	26 (2.1%)	7 (63.6%)	21 (1.7%)	6 (54.5%)
B	1202	45	104 (8.7%)	27 (60%)	71 (5.9%)	19 (35.2%)
C	512	8	43 (8.4%)	7 (87.5%)	32 (6.3%)	6 (75%)
D	495	3	45 (9.1%)	3 (100%)	28 (5.7%)	3 (100%)
E	376	5	99 (26.3%)	5 (100%)	79 (21.0%)	5 (100%)
F	489	12	106 (21.7%)	11 (91.7%)	84 (17.2%)	10 (83.3%)
Production type						
Dairy	2619	34	345 (13.2%)	34 (100%)	265 (10.1%)	32 (94.1%)
Beef	1680	50	78 (4.6%)	26 (52%)	50 (3.0%)	17 (34%)
Age interval						
< 1 year	422	64	42 (10.0%)	26 (40.6%)	28 (6.6%)	19 (29.7%)
1–4 years	2000	82	246 (12.3%)	47 (57.3%)	189 (9.5%)	38 (46.3%)
4–7 years	1209	78	106 (8.8%)	32 (41.0%)	71 (5.9%)	23 (29.5%)
> 7 years	668	72	29 (4.3%)	17 (37.5%)	27 (4.0%)	16 (22.2%)
Herd size						
< 30 animals	672	41	63 (9.4%)	22 (53.7%)	40 (5.6%)	15 (36.6%)
30–60 animals	78	18	125 (16.0%)	17 (94.4%)	97 (12.4%)	14 (77.8%)
60–100 animals	891	12	133 (15.0%)	10 (83.3%)	108 (12.1%)	10 (83.3%)
> 100 animals	1955	13	102 (5.2%)	11 (84.6%)	70 (3.6%)	10 (76.9%)
NTM/PTB presence^c^						
Yes	524	8	63 (12.0%)	7 (87.5%)	52 (9.9%)	6 (75%)
Total	4299	84	423 (9.8%)	60 (71.4%)	315 (7.3%)	49 (58.3%)

^a^OD_bovis_–OD_PBS_ ≥ 0.1 and OD_bovis_–OD_avium_ ≥ 0.1

^b^S/P ≥ 35%

^c^Herds without notification of NTM/PTB where not considered in the table

The lowest number of reactors was found in region A (2.1% for Bovigam and 1.7% for IDvet); regions B, C and D yielded a similar proportion of positive animals (ranging between 8.4–9.1% for Bovigam and 5.7–6.3% for IDvet), while the highest number of reactors was observed among samples collected from regions E and F (> 17% for both kits) (Table 3).

Dairy animals were more likely to test positive, with 2.9 and 3.4 times more reactors compared with beef cattle when considering the Bovigam and IDvet kits, respectively (Table 3). Likewise, at least one positive result to Bovigam and IDvet was found in all and 32/34 dairy herds, respectively, compared with 26/50 and 17/50 beef herds with at least one positive for Bovigam and IDvet, respectively. Finally, more reactors were also observed among animals from 1 to 4 years while fewer were found among older (> 7 years) animals irrespective of the kit (Table 3).

In addition, all 10 reactors to the PTB ELISA were negative to both IGRA kits. Also, of the 44 comparative-inconclusive animals in previous skin tests, only three animals from three herds and five animals from five herds were positive to IDvet and Bovigam, respectively, and there was only one positive to Bovigam out of the two inconclusive animals in region F.

### Agreement and correlation between test results

**Figure 2:**
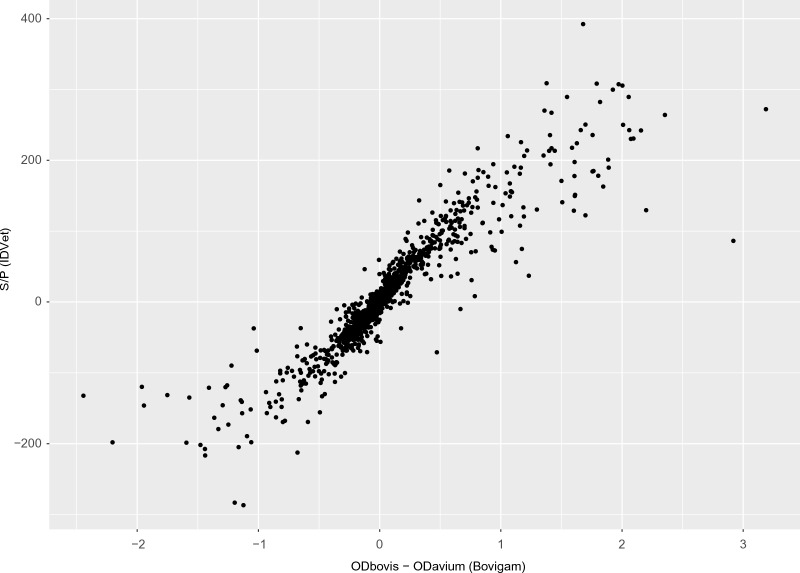
**Quantitative results for each of the samples analysed for both kits**

When the quantitative results obtained in both tests were compared, a high correlation between the S/P ratio (IDvet) and the difference between bovine and avian OD values (Bovigam) was observed (0.919, 95% CI 0. 914–0.923) (Figure 2).

The agreement between the qualitative results obtained using the default cut-offs was moderate considering both tests aim at the same target (Kappa = 0.80; 95% CI 0.76–0.83) with a significantly (McNemar test, *p* < 0.001) larger proportion of animals positive only to the Bovigam kit (Table 4).

**Table 4:** **Agreement between the results obtained in Bovigam and IDvet IFN-γ kit at default cut-off points**

		IDvet (S/P ≥ 35%)		
		Negative	Positive	Total
Bovigam (OD_bovis_–OD_PBS_ ≥ 0.1 and OD_bovis_–OD_avium_ ≥ 0.1)	Negative	3861	15	3876
	Positive	123	300	423
	Total	3984	315	4299

### ROC analysis

**Figure 3:**
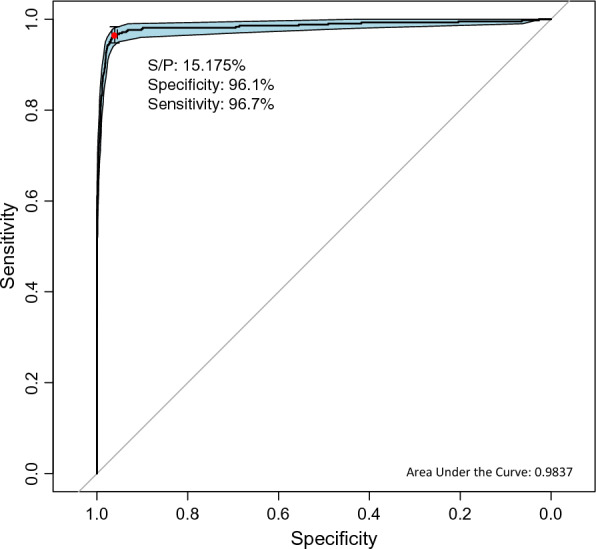
**ROC curve of the performance of the IDvet kit against the result of Bovigam kit.** Red dot represents the optimal cut-off point for maximum specificity and sensitivity (S/P = 15.175%) along with the specificity (96.1%) and sensitivity (96.7%)

The ROC analysis of the quantitative S/P values from IDvet using the qualitative results in the Bovigam kit as a reference yielded a high value of the Area Under the Curve (AUC) (0.984, 95% CI 0.975–0.992) with an optimal cut-off point of 15.175, leading to a sensitivity of 96.7% and specificity of 96.1% (Figure 3). The impact of using alternative cut-offs in the interpretation of the IDvet results in the sensitivity and specificity of the test with regards to the Bovigam results is shown in Additional file 1.

**Figure 4:**
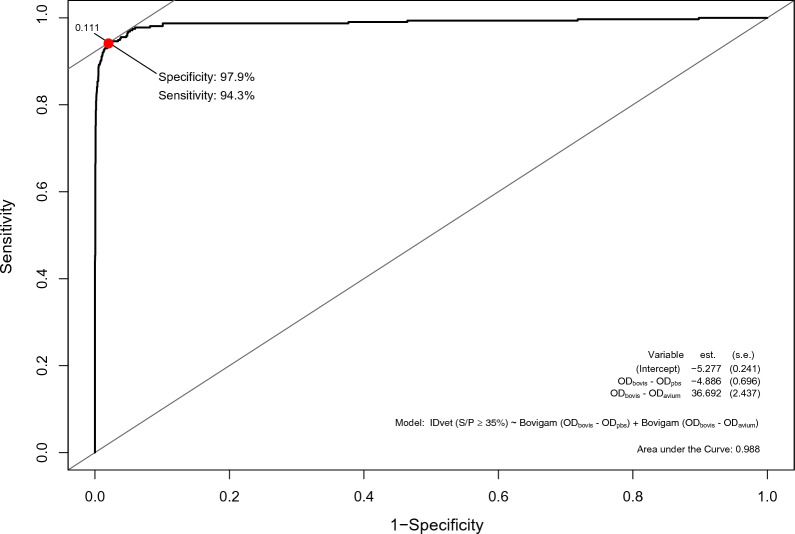
**ROC curve of the performance of the Bovigam kit against the result of IDvet kit.** On the bottom-left are represented the model coefficients for OD_bovis_–OD_PBS_ and OD_bovis_–OD_avium_. Red dot represents the logistic regression estimate for the optimal cut-off points for maximum specificity and sensitivity along with the specificity (97.9%) and sensitivity (94.3%). Optimal cut-off points are shown in Additional file 1

Likewise, the analysis of the quantitative Bovigam values (OD_bovis_–OD_PBS_ and OD_bovis_–OD_avium_) using the qualitative IDvet results as the reference revealed a high AUC value (0.988, 95% CI 0.986–0.990) with the optimal cut-off points identified, yielding a Se of 94.3% and a Sp of 97.9% (Figure 4). Additional information on the impact of other cut-offs in the Se and Sp of the Bovigam test is shown in Additional file 1.

### Multivariable regression

The final model for both kits included the age, production type and region (Table 5).

**Table 5:** **Estimates of the association of covariables with positivity according to the Bayesian logistic regression models**

Variables	Bovigam (OD_bovis_–OD_PBS_ ≥ 0.1 and OD_bovis_–OD_avium_ ≥ 0.1)			IDvet (S/P ≥ 35%)		
	Median	95% PPI^a^		Median	95% PPI^a^	
		2.5%	97.5%		2.5%	97.5%
Region^b^						
A	1	–	–	1	–	–
B	5.4	2.7	11.5	4.6	1.8	12.4
C	9.7	3.9	25.4	14.2	4.3	51.4
D	3.9	1.3	11.4	2.7	0.7	11.3
E	11.4	4.6	28.7	10.9	3.4	36.4
F	11.0	5.1	24.3	9.6	3.5	26.7
Production type^b^						
Beef	1	–	–	1	–	–
Dairy	3.7	2.1	6.7	6.3	3.0	14.3
Age^c^						
> 7 years	1	–	–	1	–	–
< 1 year	3.4	2.0	5.9	2.4	1.3	4.4
1–4 years	2.4	1.5	3.7	1.8	1.1	2.8
4–7 years	1.5	0.96	2.4	0.95	0.6	1.6

^a^Posterior probability interval

^b^Herd level

^c^Animal level

The region was strongly associated with the probability of testing positive to the test, with animals from all regions but D having a higher probability of being a reactor compared with the reference region (A) (Table 5). In addition, odds of positivity in dairy cattle were 3.7 (95% posterior probability interval (PPI): 2.1–6.7) and 6.3 (95% PPI: 3.0–14.3) higher than in beef cattle to the Bovigam and IDvet test, respectively (Table 5).

Finally, younger animals (< 1–4 years) had higher odds of being positive compared to older animals irrespective of the kit used (Table 5).

### Assessment of alternative cut-off points

**Figure 5:**
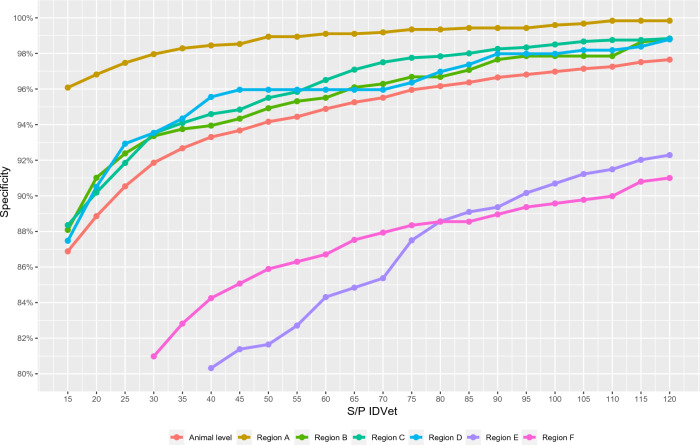
**Variation of the animal-level specificity depending on the cut-off point for IDvet (S/P ratio).** Red line represents the global animal-level specificity while the others represent region animal-level specificity

To assess the potential impact of using different cut-offs, the proportion of reactors observed when the cut-off was set at any point in the S/P ≥ 15–120% range (IDvet) and OD_bovis_–OD_PBS_ ≥ 0.05–1.0 and OD_bovis_–OD_avium_ ≥ 0.05–1.0 (Bovigam) was calculated. A perfect specificity (i.e., no reactors) was not achieved in any region regardless of the cut-off point in the ranges considered for both kits, except if we consider beef population from region A, in which a 100% specificity was achieved at a S/P ≥ 60% (IDvet) and at OD_bovis_–OD_PBS_ ≥ 0.2 and OD_bovis_–OD_avium_ ≥ 0.5 (Bovigam) (Figure 5 and Additional file 2). Furthermore, the proportion of reactors at the different cut-off values considered varied largely depending on the region (Figure 5), and for those regions in beef and dairy cattle were tested, depending on the production type within a region (see Additional file 2).

## Discussion

The great efforts invested for decades in surveillance, control and eradication programs in many countries have led to the achievement of OTF status in multiple regions and countries [36, 37]. However, in order to maintain such disease-free status, continuous monitoring is still required. In this context, the use of tests that have an optimal specificity (while maintaining an adequate sensitivity) is of paramount importance to avoid false-positive results, which could occur even with very specific tests when applied to large populations. In Europe, the single and comparative skin tests have been routinely used for this purpose, yielding excellent results in terms of specificity in the majority of the cases [38, 39]. Nevertheless, the numerous limitations associated with their use related to difficulties in their standardization (due to practical constrains in the field, the inherent subjectivity interpreting the test, and other factors linked with the test itself) [40, 41] have led to the consideration of IGRAs as an alternative for granting and maintenance of the OTF status of herds and for the intra-Community trade of animals [19]. The use of IGRAs would solve certain practical issues, since they only require a single visit to the farm, and most of the IGRA protocol is conducted in the laboratory, where conditions are easier to standardize [42]. Still, certain factors can still affect its performance [15, 18, 20], among which the cut-off value for interpretation is a major issue.

Because IGRAs in Europe have been mostly applied in bTB-infected herds, cut-off points used routinely in the EU have been typically evaluated in terms of their usefulness to maximize the diagnostic sensitivity when used in parallel to the skin tests [12, 43]. Furthermore, the specificity of IGRAs in that situation has been sometimes criticized, with most estimates suggesting it may be considerably lower than that of skin tests, although this would be also highly dependent (in addition to the cut-off) on the antigens used [23, 44, 45] and the animal populations tested [17, 18]. In this study, we aimed at assessing the performance of IGRA in OTF populations using the cut-offs currently recommended by the manufacturers on cattle populations from different regions and production types while standardizing as much as possible the protocol in order to minimize the possible impact of factors associated with the test.

Only five studies have assessed the performance of IGRAs in OTF populations, of which two were published over 15 years ago and four considered only the Bovigam kit [13–17, 22]. Overall, Bovigam specificity values obtained here were similar to previously estimated, with values around 90% despite considering different cut-offs and protocols, except for Keck et al. [17] where a 99.9% specificity was observed on bullfighting cattle, a population not evaluated here that is known to have a lower IFN-γ production [46], and for Faye et al. [22] for which depending on the interpretation criteria a 97.6–99.4% specificity was observed. In contrast, for IDvet, evaluated in OTF herds in only one study [16], previous specificity estimates were higher than the ones observed here for the overall population but very similar to those from region A, with values around 98%.

The diagnostic specificity of Bovigam and IDvet kits has been simultaneously assessed in only two studies (one in OTF herds and one in infected herds), both suggesting that the use of Bovigam would result in a higher number of reactors compared to IDvet [12, 16], similar to what was observed here (Table 3). Despite these results, the probability of yielding a positive outcome for both tests at default cut-offs was influenced by the same variables (Table 5) and, as shown by the ROC analysis from this study (Figures. 3, 4) and previous results from infected herds [12], both tests behaved similarly. Overall, this suggests that both tests are subjected to a similar effect of external variables, and that part of the differences in their performance observed here are derived from the application non-equivalent cut-off points rather than from factors such as the use of twice fold more plasma for Bovigam than IDvet, considering that both tests were performed using same PPDs, so the disparities in terms of diagnostic accuracy might not be as high as proposed between kits [47].

The influence of production type and age on the increase of the probability of observing a (false) positive result in the test identified here agrees with previous studies: dairy cattle were also more prone to yield IGRA positive results compared to beef in a previous study conducted in Italy [15], what could be attributed to exposure to other infections more prevalent in dairy animals like PTB, leading to an increased amount of non-specific immune reactions [48].

Also, we found that the risk of positivity decreased with age, with animals of < 1 year having the higher odds, as suggested by Keck et al. [17]. In contrast, this was different from the lack of an age-associated risk described in Cagiola et al. in an OTF population, although only animals between 2 and 6 years were considered there [15]. Furthermore, an increase in the risk of positivity with increasing age was suggested in another study when comparing animals of ≥ 3 years with those < 1 year [18]. Altogether, this suggests that the direction of the age effect may be different depending on local factors. For example, in our study there were no reactors of < 1 year in region D to any of the tests, and less compared to ≥ 7 years animals in dairy herds from region B (see Additional file 3). Despite this, the model indicates that < 1 year old animals have 3.4 and 2.4 more risk of positivity than older animals (for Bovigam and IDvet, respectively), what could be related to a higher non-specific IFN-γ production mediated by NK cells in younger cattle [49, 50], limiting its use in calves < 6 months old in the EU (EU-RL Standard Operating Procedure SOP/004/EURL and SOP/006/EURL).

Neither herd size nor the presence of NTM or PTB were included in the final model. Regarding the former, herd size did not influence the individual risk of being positive to any of the tests, suggesting that practices associated with larger herds (e.g., more animal movements and contacts between animals) may not play a role in such effect once other local factors are taken into account.

False-positive reactions in all bTB tests (including IGRAs) have been linked to the presence of NTM and/or PTB [51–53]. We did not find evidence of this association, but this result should be interpreted with care since herds were not subjected to a systematic evaluation of the presence of NTM or PTB; therefore, even though this variable was not included in the final model, the presence of NTM/PTB as a possible source of false-positive reactions should be further considered, and even more considering the higher prevalence of these types of infections in dairy cattle [48], which was found to play an important role in the risk of an animal testing positive for IGRA. In this sense, the use of defined MTBC-mycobacterial antigens (e.g., ESAT6, CFP10, Rv-3615c) could be useful to minimize cross-reactions in the IGRA due to NTM/PTB [22, 45, 54].

As stated before, region had a strong influence on the risk of positivity; this was also evident when changes in the proportion of reactors depending on the cut-off applied for each region were assessed (Figure 5): in certain regions (particularly region A) the use of IGRA in OTF herds could lead to high specificity values (> 98%) at cut-offs below S/P = 35%, while this could not be achieved in others (E and F) even when considering cut-offs that would most likely lead to unacceptable diagnostic sensitivities. Interestingly, these differences were observed despite using the same tuberculin for stimulation of all the blood samples regardless of their origin, thus removing the variability associated with the use of different tuberculins in different countries, a well-known factor influencing bTB diagnostic performance [55]. Season could influence the performance of the IFN-γ due to the possible impact of environmental conditions on the viability of the samples [21, 56] and the occurrence of non-specific immunological stimuli [57]. All samples were collected between November and February except those from region E, in which animals were sampled between May and June. Therefore, although a possible effect of the environmental conditions cannot be ruled out (particularly for region E), this is unlikely to explain the wide variation observed in the proportion of reactors depending on the region. Overall, no single (usable) cut-off that would yield the same specificity across populations was identified, a key aspect for its harmonisation at the EU level [19]. In this context and considering the widely different results obtained in the different regions, it would be advisable to establish the baseline reactivity of OTF populations before the implementation of the IGRA as a routine test for maintenance of the OTF status.

The use of IGRA has several advantages over the skin test, the main one being the application of an objective criteria for interpretation of the results, thus minimizing possible biases associated with external factors that can hamper accurate skinfold thickness measurements. However, in light of our results, serial application of the single or comparative skin test in animals testing positive to IGRA could help to ensure an adequate specificity if overall sensitivity is ensured, while maximizing these practical advantages.

The proportion of reactors found when using both IGRA kits evaluated here was highly dependent on the population tested, and results obtained in both kits were influenced by the age and production type of the animals to a similar degree. When considering the quantitative results both kits performed similarly, suggesting that the differences in the proportion of reactors (higher in the case of Bovigam compared to IDvet) were partly due to the use of non-equivalent cut-offs. Based on the information presented here, IGRAs may be considered a reliable alternative to skin tests in certain populations for granting and maintenance of the OTF status and movement of cattle within the EU, but no single cut-off yielded a sufficiently high specificity in all OTF populations evaluated here. Therefore, a careful preliminary assessment of the baseline IGRA reactivity in OTF populations before its application, and the possible use of other tests contemplated in the legislation (i.e., the single or comparative skin test) applied in series to IGRAs in certain epidemiological scenarios so that the overall sensitivity is not compromised, can help to ensure its adequate performance.

## Supplementary Information

**Additional file 1: Impact of different cut-offs for Bovigam and IDvet in the sensitivity and specificity of the opposite test.**

**Additional file 2: Evolution of the animal-level specificity of the Bovigam and IDvet kit depending on the cut-off point (OD or S/P ratio) used to define positivity, depending on different covariables.**

**Additional file 3: Distribution of reactors depending on the age by region and production type.**

## Data Availability

The datasets used during the current study are available from the corresponding author on reasonable request.
