# Supplementary material for: Evaluation of the performance of the IFN-γ release assay in bovine tuberculosis free herds from five European countries
Source: Vet Res. 2023 Jul 4;54:55. doi: 10.1186/s13567-023-01187-5 (PMC10320920; doi:10.1186/s13567-023-01187-5)
Supplement: Supplementary file 1 — Additional file 1: Impact of different cut-offs for Bovigam and IDvet in the sensitivity and specificity of the opposite test. [file 13567_2023_1187_MOESM1_ESM.html]

Additional file 1


# Additional file 1

### Impact of different cut-offs for Bovigam and IDvet in the sensitivity and specificity of the opposite test

#### Gómez Buendía A. et al. 2023

## Change of the relative Sensitivity & Specificity of IDvet using Bovigam ODbovis – ODPBS ≥ 0.1 & ODbovis – ODavium ≥ 0.1 as gold standard

### The optimal cut-off point is where the 3 lines intersect (S/P = 15.175%)

## Change of the relative Sensitivity & Specificity of Bovigam using IDvet S/P ≥ 35% as gold standard

### The optimal cut-off points are where the 3 layers intersect

### Optimal cut-off points for the assessment of quantitative Bovigam results in relation to qualitative IDvet results

Variation of the best optimal cut-off point for Bovigam to predict IDvet results (S/P = 35%). On the right the how a variation of the ODbovis – ODPBS affects to the ODbovis – ODavium, and vice versa on the right

| ODbovis – ODPBS | ODbovis - ODavium | ODbovis - ODavium | ODbovis – ODPBS |
| --- | --- | --- | --- |
| 0.000 | 0.082 | 0.000 | -0.614 |
| 0.025 | 0.085 | 0.025 | -0.426 |
| 0.050 | 0.088 | 0.050 | -0.238 |
| 0.075 | 0.092 | 0.075 | -0.051 |
| 0.100 | 0.095 | 0.100 | 0.137 |
| 0.125 | 0.098 | 0.125 | 0.325 |
| 0.150 | 0.102 | 0.150 | 0.513 |
| 0.175 | 0.105 | 0.175 | 0.700 |
| 0.200 | 0.108 | 0.200 | 0.888 |
