# Supplementary material for: Evaluation of the performance of the IFN-γ release assay in bovine tuberculosis free herds from five European countries
Source: Vet Res. 2023 Jul 4;54:55. doi: 10.1186/s13567-023-01187-5 (PMC10320920; doi:10.1186/s13567-023-01187-5)
Supplement: Supplementary file 2 — Additional file 2: Evolution of the animal-level specificity of the Bovigam and IDvet kit depending on the cut-off point (OD or S/P ratio) used to define positivity, depending on different covariables. [file 13567_2023_1187_MOESM2_ESM.html]

Additional file 2


# Additional file 2

### Evolution of the animal-level specificity of the Bovigam and IDvet kit depending on the cut-off point (OD or S/P ratio) used to define positivity, depending on different covariables

#### Gómez Buendía A. et al. 2023

## Region

| Region | Total |
| --- | --- |
| A | 1225 |
| B | 1202 |
| C | 512 |
| D | 495 |
| E | 376 |
| F | 489 |
| Total | 4299 |

- ID Screen® Ruminant IFN-g (IDvet)

- BovigamTM TB Kit (Bovigam)

## Age

| Age | Total |
| --- | --- |
| 0 - 1 years | 422 |
| 1 - 4 years | 2000 |
| 4 - 7 years | 1209 |
| 7 - 19 years | 668 |
| Total | 4299 |

- ID Screen® Ruminant IFN-g (IDvet)

- BovigamTM TB Kit (Bovigam)

## Aptitude

| Aptitude | Total |
| --- | --- |
| Beef | 1680 |
| Dairy | 2619 |
| Total | 4299 |

- ID Screen® Ruminant IFN-g (IDvet)

- BovigamTM TB Kit (Bovigam)

## Region & Aptitude

| Region | Beef | Dairy | Total |
| --- | --- | --- | --- |
| A | 576 | 649 | 1225 |
| B | 595 | 607 | 1202 |
| C | 400 | 112 | 512 |
| D | 0 | 495 | 495 |
| E | 0 | 376 | 376 |
| F | 109 | 380 | 489 |
| Total | 1680 | 2619 | 4299 |

## Age & Aptitude

| Age | Beef | Dairy | Total |
| --- | --- | --- | --- |
| 0 - 1 years | 231 | 191 | 422 |
| 1 - 4 years | 611 | 1389 | 2000 |
| 4 - 7 years | 406 | 803 | 1209 |
| 7 - 19 years | 432 | 236 | 668 |
| Total | 1680 | 2619 | 4299 |

## Region A - Age & Aptitude

| Age | Beef | Dairy | Total |
| --- | --- | --- | --- |
| 0 - 1 years | 100 | 53 | 153 |
| 1 - 4 years | 124 | 343 | 467 |
| 4 - 7 years | 119 | 203 | 322 |
| 7 - 19 years | 233 | 50 | 283 |
| Total | 576 | 649 | 1225 |

## Region B - Age & Aptitude

| Age | Beef | Dairy | Total |
| --- | --- | --- | --- |
| 0 - 1 years | 71 | 54 | 125 |
| 1 - 4 years | 240 | 349 | 589 |
| 4 - 7 years | 152 | 162 | 314 |
| 7 - 19 years | 132 | 42 | 174 |
| Total | 595 | 607 | 1202 |

## Region C - Age & Aptitude

| Age | Beef | Dairy | Total |
| --- | --- | --- | --- |
| 0 - 1 years | 42 | 3 | 45 |
| 1 - 4 years | 189 | 71 | 260 |
| 4 - 7 years | 103 | 26 | 129 |
| 7 - 19 years | 66 | 12 | 78 |
| Total | 400 | 112 | 512 |

## Region D - Age & Aptitude

| Age | Beef | Dairy | Total |
| --- | --- | --- | --- |
| 0 - 1 years | 0 | 19 | 19 |
| 1 - 4 years | 0 | 242 | 242 |
| 4 - 7 years | 0 | 189 | 189 |
| 7 - 19 years | 0 | 45 | 45 |
| Total | 0 | 495 | 495 |

## Region E - Age & Aptitude

| Age | Beef | Dairy | Total |
| --- | --- | --- | --- |
| 0 - 1 years | 0 | 0 | 0 |
| 1 - 4 years | 0 | 245 | 245 |
| 4 - 7 years | 0 | 117 | 117 |
| 7 - 19 years | 0 | 14 | 14 |
| Total | 0 | 376 | 376 |

## Region F - Age & Aptitude

| Age | Beef | Dairy | Total |
| --- | --- | --- | --- |
| 0 - 1 years | 18 | 62 | 80 |
| 1 - 4 years | 58 | 139 | 197 |
| 4 - 7 years | 32 | 106 | 138 |
| 7 - 19 years | 1 | 73 | 74 |
| Total | 109 | 380 | 489 |
