# Supplementary material for: Evaluation of the performance of the IFN-γ release assay in bovine tuberculosis free herds from five European countries
Source: Vet Res. 2023 Jul 4;54:55. doi: 10.1186/s13567-023-01187-5 (PMC10320920; doi:10.1186/s13567-023-01187-5)
Supplement: Supplementary file 3 — Additional file 3: Distribution of reactors depending on the age by region and production type. [file 13567_2023_1187_MOESM3_ESM.pdf]

Additional file 3

Distribution of reactors depending on the age by region and production type.

Gomez-Buendia A. et al. 2023

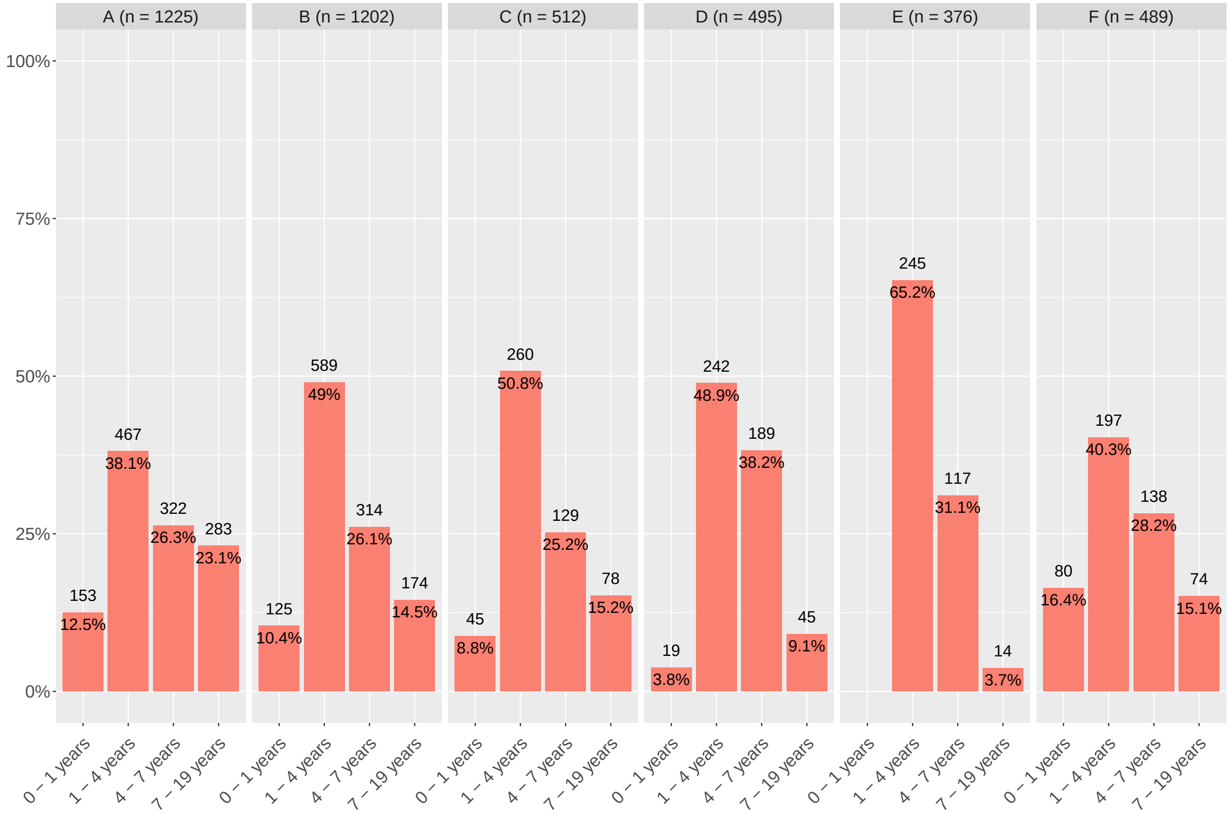

Additional Figure 3.1 Distribution of the age grouped in four categories by region of origin

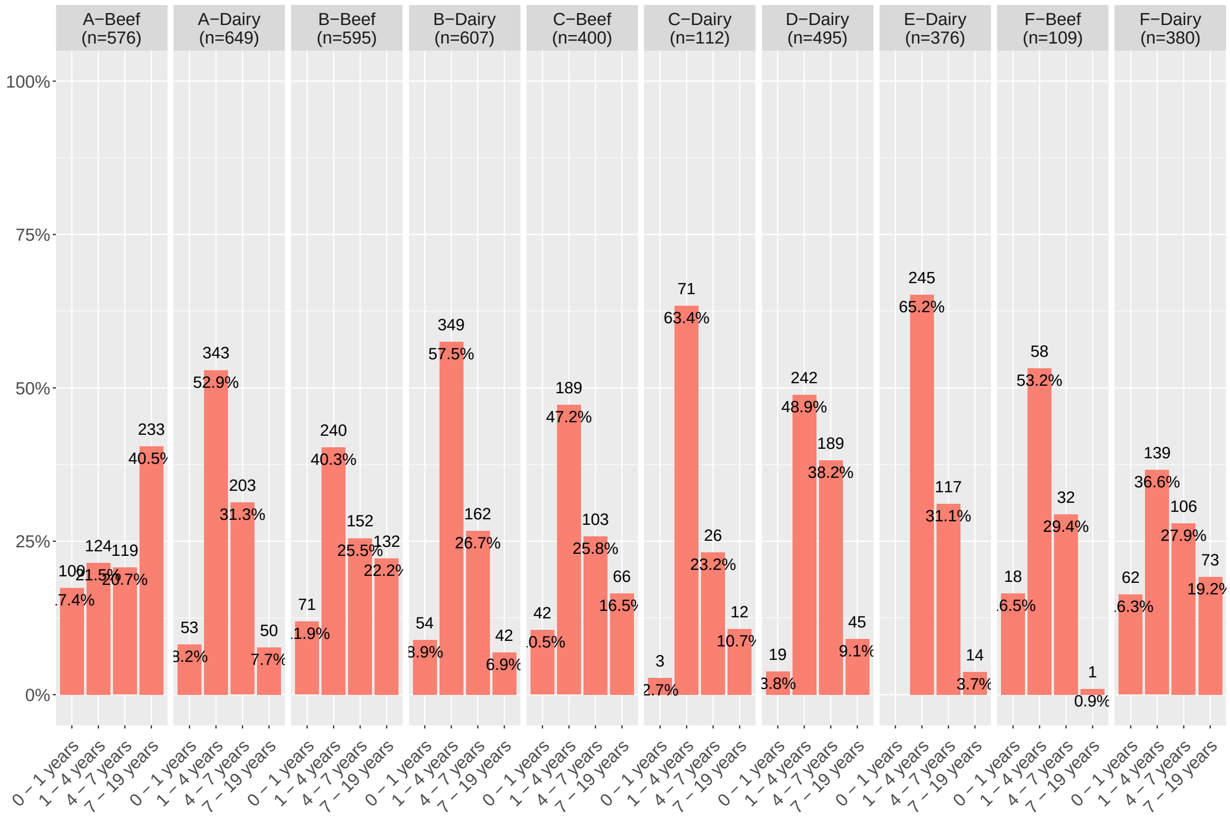

Additional Figure 3.2 Distribution of the age grouped in four categories by region of origin and production type

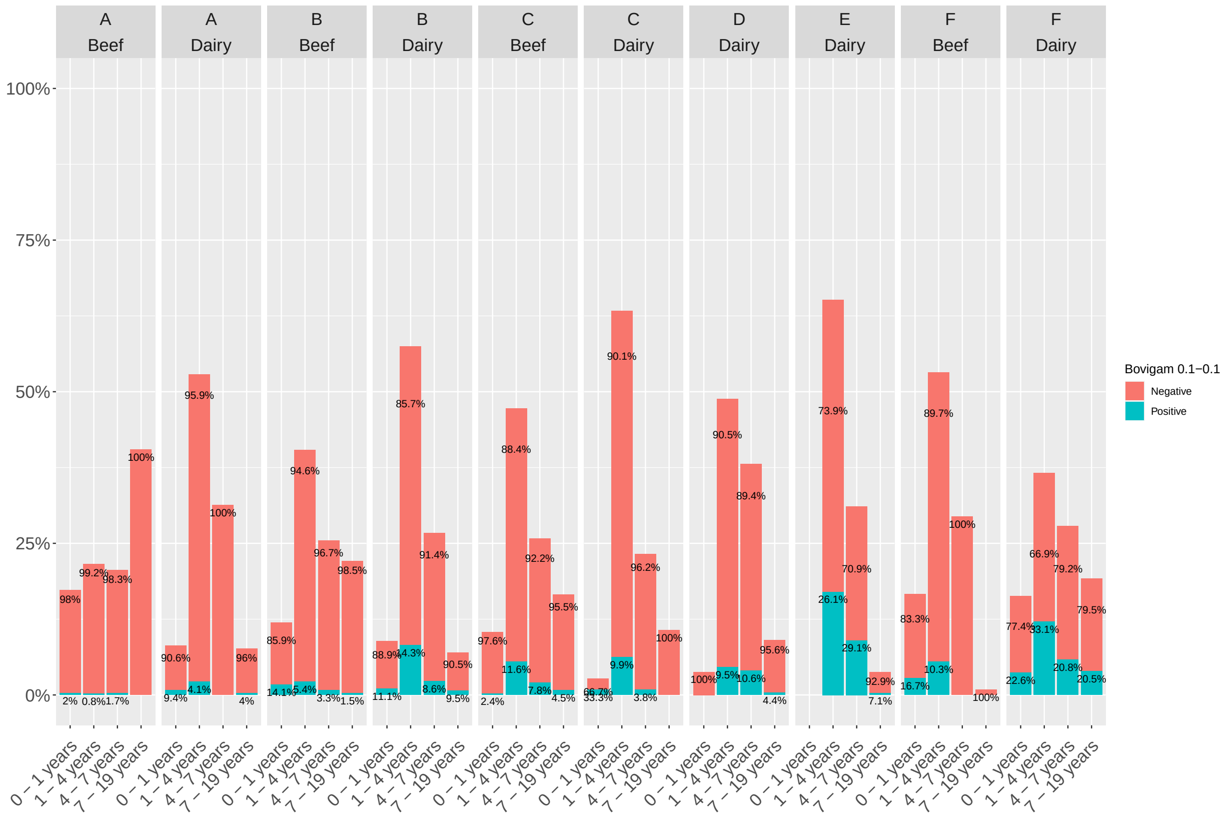

Additional Figure 3.3 Percentage of reactors to Bovigam kit (cut-off point of $ODbovine - avian \geq 0.1$ & $ODbovine - PBS \geq 0.1$) by region, production type and age.

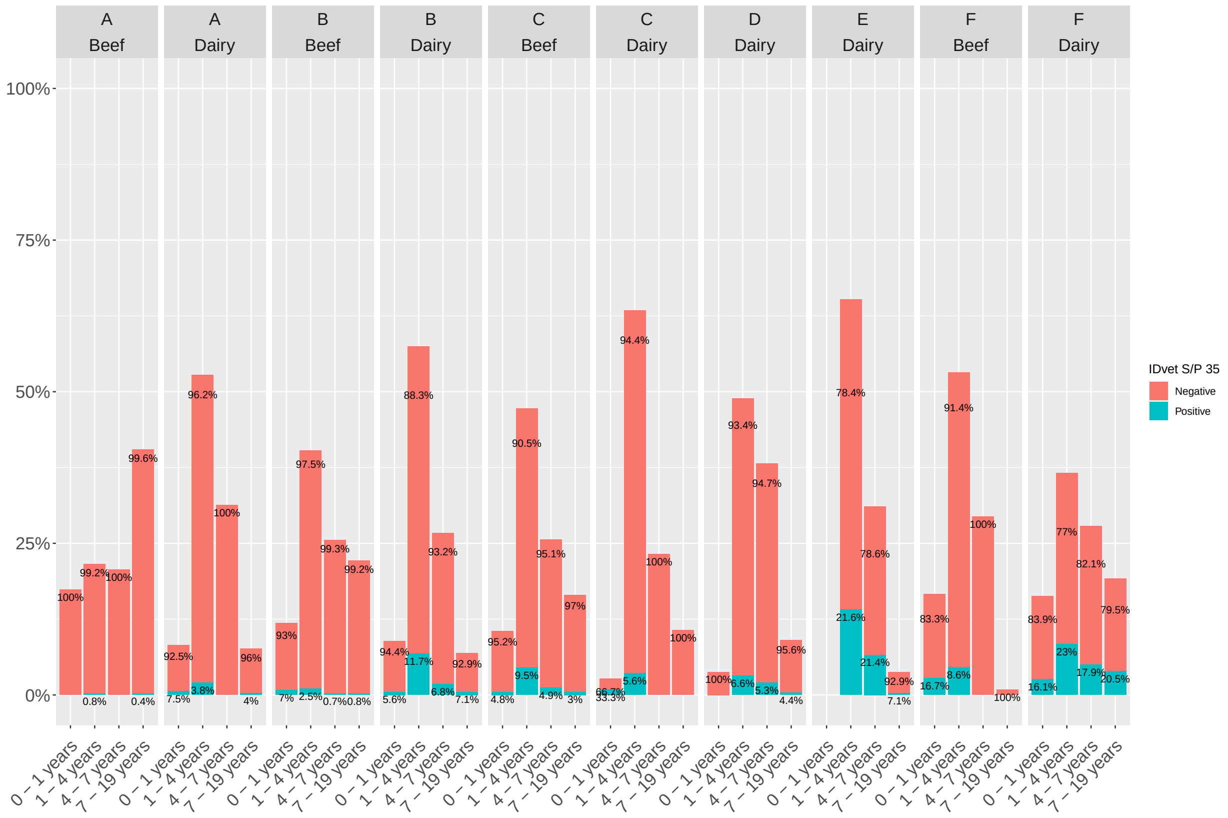

Additional Figure 3.4 Percentage of reactors to IDvet kit (cut-off point of $S/P \geq 35$) by region, production type and age.
